# Dual Roles of the Lysine-Rich Matrix Protein (KRMP)-3 in Shell Formation of Pearl Oyster, *Pinctada fucata*

**DOI:** 10.1371/journal.pone.0131868

**Published:** 2015-07-10

**Authors:** Jian Liang, Guangrui Xu, Jun Xie, Ilsun Lee, Liang Xiang, Hongzhong Wang, Guiyou Zhang, Liping Xie, Rongqing Zhang

**Affiliations:** 1 Institute of Marine Biotechnology, Collaborative Innovation Center of Deep Sea Biology, School of Life Sciences, Tsinghua University, Beijing, China; 2 Protein Science Laboratory of the Ministry of Education, Tsinghua University, Beijing, China; RMIT University, AUSTRALIA

## Abstract

Matrix proteins play important roles in shell formation. Our group firstly isolated three cDNAs encoding lysine-rich matrix protein from *Pinctada fucata* in 2006. However, the functions of KRMPs are not fully understood. In addition, KRMPs contain two functional domains, the basic domain and the Gly/Tyr domain respectively. Based on the modular organization, the roles of their two domains were poorly characterized. Furthermore, KRMPs were then reported in other two species, *P*. *maxima* and *P*. *margaritifera*, which indicated that KRMPs might be very important for shell formation. In this study, the characterization and function of KRMP-3 and its two functional domains were studied *in vitro* through purification of recombinant glutathione S-transferase tagged KRMP-3 and two KRMP-3 deletion mutants. Western blot and immunofluorescence revealed that native KRMP-3 existed in the EDTA-insoluble matrix of the prismatic layer and was located in the organic sheet and the prismatic sheath. Recombinant KRMP-3 (rKRMP-3) bound tightly to chitin and this binding capacity was duo to the Gly/Tyr-rich region. rKRMP-3 inhibited the precipitation of CaCO_3_, affected the crystal morphology of calcite and inhibited the growth of aragonite *in vitro*, which was almost entirely attributed to the lysine-rich region. The results present direct evidence of the roles of KRMP-3 in shell biomineralization. The functional rBR region was found to participate in the growth control of crystals and the rGYR region was responsible to bind to chitin.

## Introduction

Mollusk shell formation is a typical biomineralization process, in which mineral crystals accumulate in a controlled manner [1–3]. Among molluscs, some bivalve species are of great commercial interest including *Pinctada fucata*, the shell of which is composed of three distinct layers: the outer organic periostracum and two other calcified layers, the prismatic and nacreous layers [4, 5]. The organic matrix makes up less than 5% of the shell and is thought to be responsible for biocrystal synthesis. Although the shell organic matrix components include proteins, polysaccharide and lipids, it is thought that proteins are the major macromolecules that control the essential phases, including crystal nucleation, crystal orientation and the determination of crystal polymorph and crystal morphology [6].

Currently, nearly 50 matrix protein sequences have been identified [6]. Most of these sequences were obtained through molecular biology methods. The evaluation of the function of the matrix proteins largely depends on their localization via *in situ* hybridization in mantle tissue and Western blot analysis. What’s more, clarification of their functions requires further *in vitro* investigations at the protein level. In addition, only a few proteins have been isolated and purified directly. Accordingly, the expression and purification of recombinant proteins *in vitro* is imperative. However, among the matrix proteins for whose full-length DNA sequences are available, only a small fraction of these proteins could be expressed. Although various protein expression systems were screened and different expression conditions were optimized, the difficulty of obtaining recombinant matrix proteins still remains.

In the last few decades, researchers have shown that matrix proteins are multifunctional, and this multifunctional activity is a result of their modular organization. For example, nacrein has a carbonic anhydrase domain and a Gly-X-Asn repeat domain. The carbonic anhydrase showed enzymatic activity and the Gly-X-Asn repeats domain inhibited crystallization [7, 8]. Another example is Lustrin A, which was isolated from *Haliotis rufescens*. This protein consists of five specific domains, ten highly conserved cysteine-rich domains, eight proline-rich domains, a glycine and serine-rich domain, a basic domain and a C-terminal domain [9]. However, the different functions of the various domains of Lustrin A were not revealed and only some conserved sequences in the domains were evaluated.

The lysine-rich matrix protein (KRMP) family members are typical basic matrix proteins cloned from the mantle of the pearl oyster, *P*. *fucata* [10]. KRMPs are rich in lysine, glycine and tyrosine amino acids. The mature KRMPs include two functional regions: a lysine-rich basic region (BR) and a Gly/Tyr-rich region (GYR). The predicted p*I* of the KRMPs is between 9.5 and 9.8. Currently, KRMPs have been found in three commercially valuable pearl oysters: *P*. *fucata*, *P*. *maxima* [11] and *P*. *margaritifera* [12, 13]. Furthermore, the KRMP mRNA expression level was found to be significantly higher than other genes, except for the shematrin matrix protein family, which indicated that KRMPs may play important roles in shell formation [12, 14]. RT-PCR and *in situ* hybridization analysis indicated that KRMPs were involved in the formation of the prismatic layer. However, the functions of KRMPs are not fully understood and a full understanding of the roles of the individual functional domains remains poorly characterized. Importantly, studies of the KRMP matrix family will not only decipher the mechanism of the interactions between proteins and CaCO_3_, but also offer interesting perspectives in the biomaterials field.

In an attempt to investigate the roles that the KRMP family might play in the biomineralization of *P*. *fucata*, we prepared recombinant GST-tagged rKRMP-3, rBR and rGYR using *E*. *coli* expression systems. We initially investigated the location of native KRMP-3 in the shell using an antibody produced against rKRMP-3. Subsequently, the purified rKRMP-3, rBR and rGYR were characterized and assessed by biochemical analysis, including a chitin binding assay, an inhibitory activity assay on calcium carbonate precipitation, and the influence on the morphology of calcite and aragonite. The results provide insight into the function of KRMP-3 and the individual contributions of its two functional domains to the activity of the protein.

## Materials and Methods

### Ethics Statement

This study was approved by the Animal Ethics Committee of Tsinghua University, China.

### Construction of the expression vectors for KRMP-3 and its two functional regions

The expression vector for the recombinant expression of KRMP-3 was prepared as follows. Live individuals of adult *P*. *fucata* were purchased from Zhanjiang, China and maintained in aerated artificial seawater (ASW) for a week. Total RNA of the mantle tissues from six individuals was extracted using the TRIzol reagent (Invitrogen, Carlsbad, CA, USA). The integrity of the RNA was determined by electrophoresis on a 1.2% formaldehyde-denatured agarose gel stained with ethidium bromide. The quality and quantity of RNA was determined by measuring the OD_260/280_ and OD_260_ with a NanoDrop Lite spectrophotometer (Thermo, Waltham, MA, USA). The extracted RNA (1 μg) was used to synthesize single-stranded cDNA with the SuperScript II RNase H-reverse transcriptase (Invitrogen) and an adaptor-oligo (dT) primer following the manufacturer’s instructions. The open reading frame of KRMP-3 (Genbank number: DQ114790) was amplified with the primer pair K5 (CCG*GAATTC*TACTGGCATAAACCTAATC) and K3 (CCG*CTCGAG*CGGTTAGTATTTGTATTTACGATGAC) containing the *EcoR I* site and the *Xho I* site (underlined), respectively. The PCR product was ligated into the pMD19-T vector and transformed into TOP10 competent cells (Transgen, Beijing, China). After a positive colony had been detected and sequenced, the rKRMP-3 insert was released from the pMD19-T vector by digestion with *EcoR I* and *Xho I*, and then subcloned into the expression vector pGEX-4T-1 (Invitrogen). The construction of vectors for the expression of BR and GYR followed a similar procedure as used to produce the rKRMP-3 expression construct. For BR, the primer pair of K5 and BR3 (CCG*CTCGAG*CGGTTAAAATTTATGAAGGCACCAT) was used to amplify the sequence, and the primer pair of GY5 (CCG*GAATTC*GGTGGGCATTATCCGTAT) and K3 was used to amplify the GYR sequence.

### Recombinant expression of KRMP-3, BR and GYR in *E*. *coli*

The above expression vectors were transformed into the BL21 (DE3) strain and after incubation at 37°C for 12 h, one individual colony for each construct was inoculate in 10 ml of LB medium containing 100 μg/ml ampicillin. After overnight incubation, each of the starting cultures was diluted 100-fold with LB medium. The diluted cultures were incubated at 37°C until the OD_600_ reached ~0.8. Isopropyl thio-β-D-galactoside was then added to the culture to a final concentration of 0.8 mM, and the temperature was reduced to 16°C. After incubation for 20 h, the bacterial cells were harvested and the cells were suspended in 60 ml cold PBS buffer (10 mM, pH 7.4) containing 5 mM DTT and 50 μg/ml PMSF and lysed by sonication on ice. After adding 1% Triton-X-100, the suspensions were centrifuged at 12,000 ×*g* for 40 min at 4°C. The supernatant was filtrated with a 0.20 μm filter (Pall, Ann Arbor, MI, USA) and prepared for affinity chromatography.

### Purification of rKRMP-3, rBR and rGYR

The GST-tagged recombinant proteins were purified by GSTrap FF columns (GE Healthcare). The columns were pre-equilibrated with three column volumes of PBS. Each sample was loaded onto the column at a rate of 0.25 ml/min and the columns were washed with PBS. Finally, rKRMP-3, rBR and rGYR were eluted with 20 mM reduced glutathione in 50 mM Tris–HCl (pH 8.0). The yield of each eluted recombinant protein was measured using a BCA assay kit (Pierce).

### Preparation of an antiserum against rKRMP-3

A rabbit polyclonal antiserum raised against purified recombinant KRMP-3 was obtained by immunizing into New Zealand rabbits according to standard immunization procedures. The anti-rKRMP-3 serum was purified according to the method of Fan [15] and applied to Western blot analysis and immunolocalization.

### Shell preparation and matrix extraction

The shell matrix is often classified as either soluble or insoluble material based on the solubility after decalcification with reagents such as EDTA. The soluble matrix was extracted according to the method reported by Miyamoto [7]. Fresh and cleaned shells of *P*. *fucata* were immersed in 5% sodium hydroxide for 12 h and subsequently rinsed in distilled water. The calcitic and aragonitic portions of the shell were then separated mechanically and crushed to fine powders (< 100 μm). Ground shell powders (100 g) were decalcified with 500 ml 0.5 M EDTA (pH 8.0) containing 0.01% sodium azide at 4°C with continuous stirring. After 4 d, a soluble extract was obtained by centrifugation at 16,000 × *g* for 30 min at 4°C, and then diluted with an equal volume of ddH_2_O and desalted by ultrafiltration (Millipore) and lyophilized. The precipitate was thoroughly rinsed with distilled water and treated with denaturing solution (30 mM Tris-HCl, pH 8.0, 1% SDS, 10 mM dithiothreitol) at 100°C for 15 min. After centrifugation at 14,000 × *g* for 20 min, the denatured samples were collected.

### Western blot analysis

The EDTA soluble and insoluble matrix samples were run on a 15% SDS-PAGE gel and transferred onto a polyvinylidene difluoride membrane. The membrane was blocked in Tris buffered saline Tween-20 (TBST) containing 5% low-fat milk overnight at 4°C. Subsequently, proteins on the PVDF membrane were detected with a 1:1000 dilution of the antibody against rKRMP-3 or the pre-immune serum and an anti-GST antibody as controls. After washing thoroughly four times with TBST, the membrane was treated with a 1:500 dilution of the horseradish peroxidase-labeled goat anti-rabbit IgG in TBST containing 5% milk for 1 h. The membrane was then washed four times with TBST and finally visualized with the 3, 3’-diaminobenzidine tetrahydrochloride (DAB) reagent kit (Tian Gen, China).

### Immunohistochemistry

After being washed and sonicated in distilled water for 10 min, a cross-section of the shell was incubated in a solution of 0.5 M EDTA (pH 8.0), containing 4% formaldehyde and 0.5% cetylpyridinium chloride (CPC) at room temperature with gentle shaking for 3 d. After complete decalcification, the samples were thoroughly washed with distilled water and blocked in 10% goat serum for 2 h. The samples were then incubated in PBS containing polyclonal antibodies produced against rKRMP-3 or the preimmune serum. In both cases, bovine serum albumin 0.25% w/v (Sigma) was used to block non-specific binding. After incubation for 2 h, unbound antibodies were removed by washing twice with PBS containing Tween 20 (0.05% w/v) for 5 min and twice with PBS for 10 min. The secondary antibody, DyLight TM 594-conjugated goat-anti-rabbit antibody (Jackson, diluted 1:1000 in PBS) was added and incubated for 40 min. Unbound secondary antibodies were washed with PBS containing Tween 20 (0.05% w/v) three times for 15 min and samples were then rinsed in water and examined by fluorescence microscopy (Leica, DMIRB).

### Assessment of chitin-binding activity

Chitin-binding activity was assayed by a modification of Inoue’s method [16]. Prior to the assay, the chitin was washed with 0.2 M HCl and 0.2 M NaOH, and equilibrated with distilled water. rKRMP-3, rBR, rGYR and GST (15 μg each) were dissolved in 20 μl 0.1% NH_4_HCO_3_, and incubated with chitin (5 mg) for 12 h at 25°C with continuous stirring. After removal of the solution, the residues were washed with 200 μl distilled water, 200 μl of 0.2 M NaCl and 1 M acetic acid. The final component was boiled in 30 μl of 2% SDS containing 20% β-mercaptoethanol for 15 min. The supernatants of each washing step were concentrated and subjected to SDS-PAGE.

### *In Vitro* growth of crystals in the presence of rKRMP-3, rBR and rGYR

The effect of rKRMP-3, rBR and rGYR on the morphology of calcite and aragonite crystals was tested by incubating recombinant protein and a freshly-prepared saturated solution of calcium carbonate on a siliconized cover glass at 20°C with or without magnesium chloride. The saturated calcium carbonate solution was prepared according to the method of Yan [17]. Crystallization experiments were carried out with increasing amounts of rKRMP-3, rBR and rGYR (2–40 μg/ml). The experiments were repeated three times.

### Crystal Characterization

SEM and Raman spectroscopy were used for morphological observation and identification of the induced crystals. SEM (FEI quanta 200) was performed after gold sputtering of the induced crystals at an acceleration voltage of 10–15 kV. Raman spectra of the crystals were recorded with an excitation wavelength of 514 nm provided by a Renishaw RM2000 spectrometer, the argon laser was limited to a power of 4.6 mW. The spectra were scanned for 60 s from 100 to 1500 cm^–1^.

### Assessment of inhibitory activity on calcium carbonate precipitation

The inhibitory activities of rKRMP-3, rBR and rGYR on calcium carbonate precipitation were assessed according to the method of Suzuki with minor modifications [18]. The formation of calcium carbonate precipitates was followed by the turbidity of the solution containing 100 μl 100 mM NaHCO_3_ (pH 8.4, Sigma) and 10 μl sample solution, after 100 μl 100 mM CaCl_2_ (Sigma) was added to the solution. Changes in the turbidity of the solution were monitored every minute for 5 min by measuring the absorbance at 570 nm with a Model 550 Microplate Reader (Bio-Rad). Results were repeated four times at every minute and presented as the mean ± SD.

## Results and Discussion

### Expression and purification of rKRMP-3, rBR and rGYR

**Fig 1:**
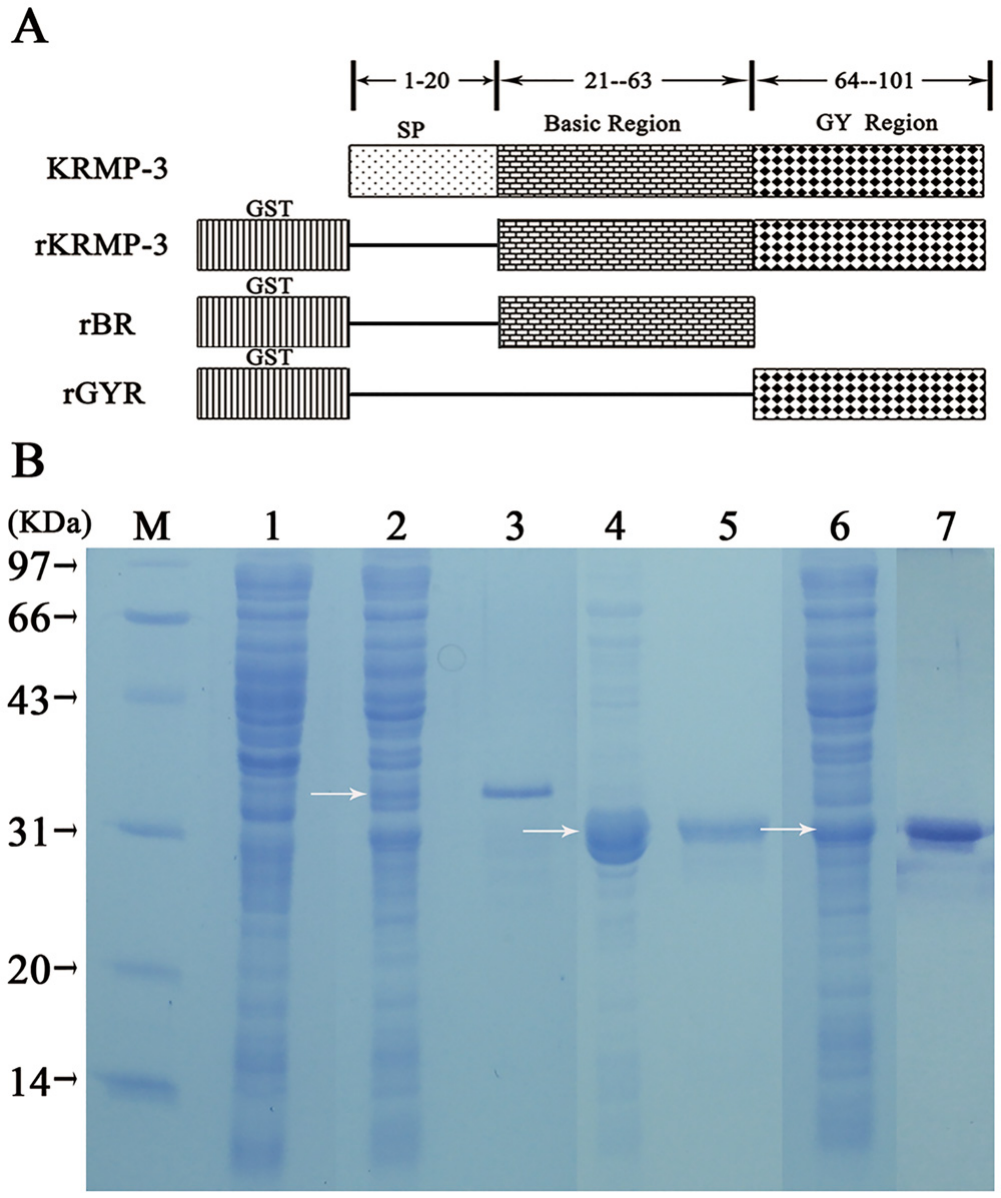
Recombinant derivatives of KRMP-3. (A) Schematic representation of KRMP-3, rKRMP-3, rBR and rGYR. KRMP-3, wild-type full-length KRMP-3 containing the signal peptide (SP) sequence and consisting of 101 amino acid residues; rKRMP-3, KRMP-3 devoid of the SP sequence and contains 81 amino acid residues and its N-terminally tagged with an affinity GST; rBR, the basic region of KRMP-3 containing 43 amino acid residues (21–63) and N-terminally tagged with an affinity GST; rGYR, the Gly/Tyr-rich region of KRMP-3 containing the C-terminal 38 amino acid residues (64–101) and N-terminally tagged with GST as well. (B) Expression and purification of recombinant KRMP-3, GYR and BR in *E*. *coli*. Arrows represent the induced proteins by IPTG. M: protein molecular mass markers; lane 1: uninduced whole-cell lysate; lane 2, 4, 6: whole-cell lysate of rKRMP-3, rGYR and rBR induced by 0.8 mM IPTG; lane 3, 5, 7: purified rKRMP-3, rGYR and rBR eluted from GSTrap FFcolumn.

Sequence-verified pGEX-4T-1 vectors containing either the KRMP-3 or BR or GYR were used for expression in *E*. *coli* (Fig 1A). After induction with IPTG, the three GST fusion proteins were effectively expressed (Fig 1B). The major bands of rKRMP3, rBR and rGYR on the gel migrated between 30 and 43 kDa, which correspond to the molecular masses of the fusion constructs: 35 kDa (rKRMP-3), 30.8 kDa (rBR) and 30.2 kDa (rGYR) (26 kDa GST plus either 9, 4.8 or 4.2 kDa, respectively). The molecular mass of rBR is slightly higher than rGYR. We also expressed and purified GST using the same method as a negative control.

Traditionally, most matrix proteins have been isolated directly from solubilizing the shell powder using various agents, including ddH_2_O [17], PBS [19], EDTA [7], or weak dilute acids [20]. Among the isolated proteins, most were acidic matrix proteins, whereas basic matrix proteins that were usually components of the insoluble organic framework were barely solubilized by EDTA or weak dilute acids. When treated with detergents at high temperature, most basic matrix proteins isolated from the insoluble components were found to be denatured and inactive. Then the use recombinant expression of these proteins seemed to be a viable alternative to characterize matrix proteins. In our study, *P*. *pastoris* and *E*. *coli* expression systems were selected. A GST fusion KRMP-3 was successfully prepared in *E*. *coli* after attempting to express a His-tagged KRMP-3 expression system in *P*. *pastoris* and *E*. *coli* failed. The two functional regions of KRMP-3 were also expressed to investigate their functions. Following the expression and purification of the three GST fusion proteins, we tried to remove the GST tag by proteolytic digestion; however, low recovery yields of the target proteins hampered efforts to study rKRMP-3, rBR, and rGR without the GST tag. Similar issues have been reported for the preparation of recombinant matrix proteins MSI7 [21] and PFMG1 [22]. Consequently, purified rKRMP-3, rBR and rGR with the GST tag were used to evaluate the function of these three constructs *in vitro*.

### Western blot and Immunolocation of native KRMP-3 in the shell

**Fig 2:**
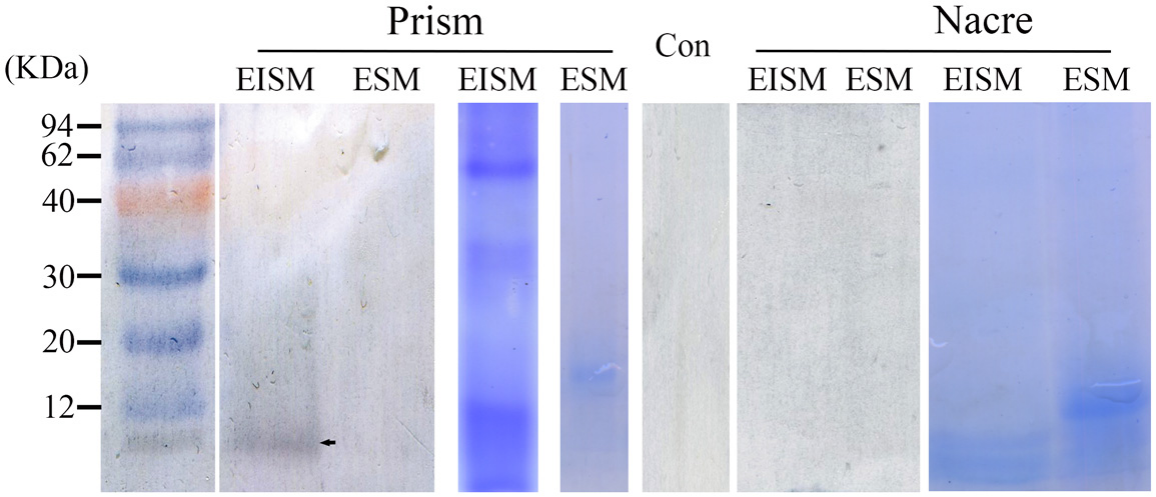
Western blot analyses using anti-rKRMP-3 antibody. The EDTA extracts from the insoluble matrices (EISM) and soluble matrices (ESM) of the prismatic layer, the EISM and ESM of the nacreous layer were applied to SDS-PAGE and subjected to western blot analysis by using anti-rKRMP-3 antibody or preimmune serum as a control. The arrow indicated that KRMP3 existed in the EISM of the prismatic layer. The con lane is the control group incubated with preimmune serum.

We examined the presence of native KRMP-3 with the anti-rKRMP-3 antibody or pre-immune serum as a negative control. Western blot analysis revealed that a single intense band (approximately 10 KDa) corresponding to native KRMP-3 was detected in the EDTA-insoluble matrix (EISM) extracts from the calcite layer, but not in the EISM extracts from the nacreous layer or the EDTA-soluble matrix (ESM) from the two layers (Fig 2).

**Fig 3:**
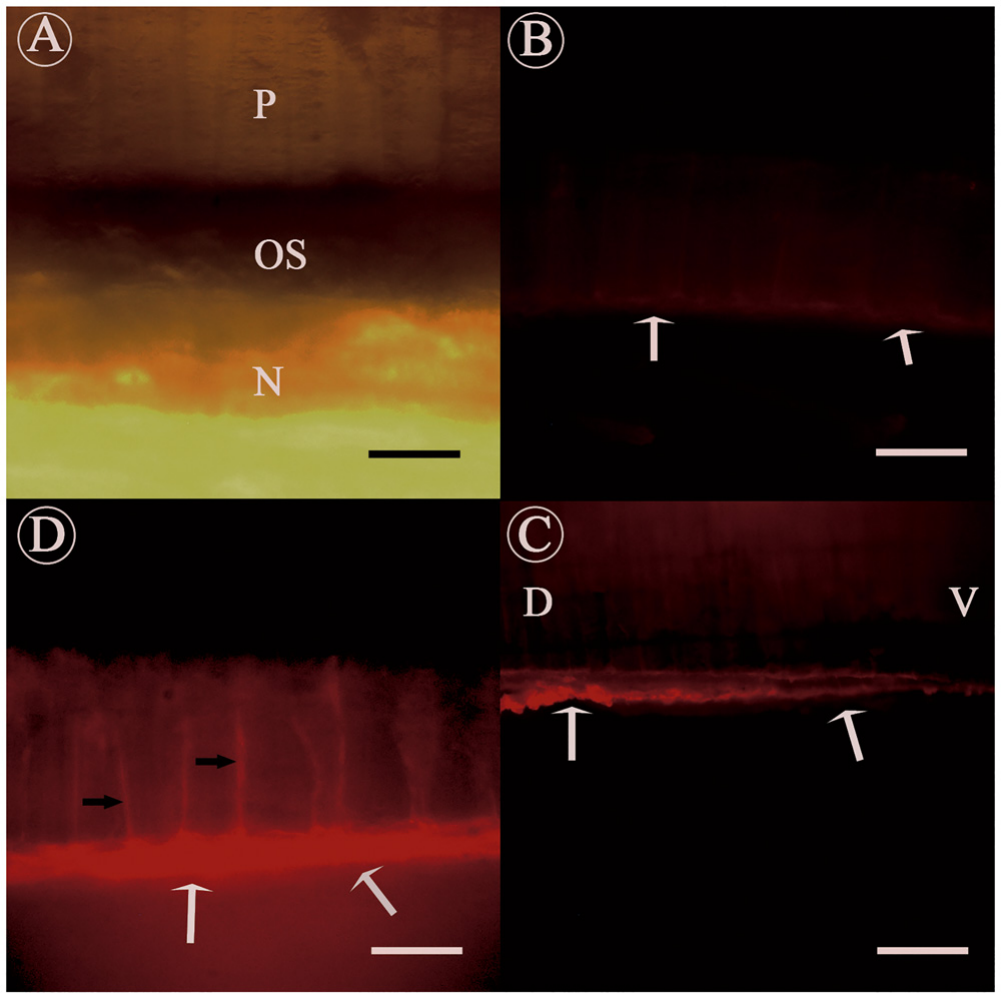
Immunofluorescence localization of native KRMP-3 in the adult shell of *P*. *fucata*, with anti-rKRMP-3 antibody. Cleaned and modified shell was decalcified and incubated using anti-rKRMP-3 antibody (C, D) or preimmune serum (B). A, Light micrograph of the shell. The shell presents a representative nacroprismatic microstructure, with columnar calcitic prisms in the upper and nacreous layer in the lower. The two calcified layers are generally separated by an organic layer. P, prismatic layer; N, nacreous layer; OS, organic sheet. B, negative control staining with preimmune serum. The white arrows indicated only a small amount of background staining was detected in the organic sheet. C, staining with anti-rKRMP-3 antibody. The white arrows showed positive signal in the organic sheet. Meanwhile, the positive signal from the ventral to dorsal reflects the distribution of organic sheet; D, dorsal; V, ventral. D, high magnification of the sample. Positive signal detected not only in the organic sheet (white arrows), but also in the prismatic sheath (black arrows). Scale bars in (A, D), 12.5 μm and in (B, C), 50 μm.

The shell of *P*. *fucata* presents a representative nacroprismatic microstructure. The outer structure is the prismatic layer and the inner structure is the nacreous layer, and between these two layers is an organic sheet (Fig 3A). The immunofluorescence localization experiments on thin transversal adult shell sections showed that native KRMP-3 existed in the organic sheet and the prismatic sheath (Fig 3C and 3D). A small amount of background staining was visible in the control sample (Fig 3B), but much weaker than the fluorescence seen in Fig 3C and 3D. From ventral to dorsal the content of KRMP-3 increased with the sheet gradually becoming thicker (Fig 3C and 3D). Immunofluorescence of several different *P*. *fucata* shells showed the same phenomenon (S1 Fig).

The results of the immunofluorescence and Western blot analyses showed that KRMP-3 was localized to the organic sheet [17, 23, 24] and the prismatic sheath [25], which was proved to be a component of the insoluble matrix. This result was consistent with the data obtained from RT-PCR and *in situ* hybridization [10]. KRMP-3 is the second matrix protein, after the discovery of Prisilkin-39, to be found in the organic layer.

### Chitin-binding ability

**Fig 4:**
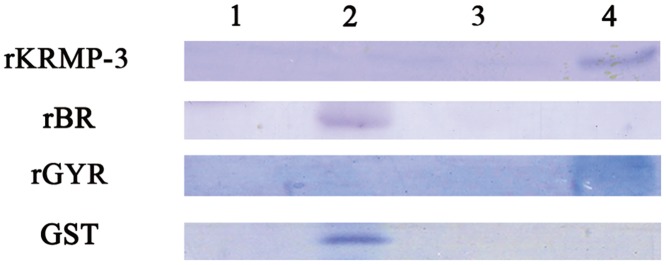
Chitin-binding assay. Lane 1, water-washings; lane 2, 0.2 M NaCl-washings; lane 3, 1 M acetic acid-washings; lane 4, extract with detergent-SDS/β-mercaptoethanol for 10 min at 100°C. GST was used as a negative control.

Chitin is the major component of the insoluble matrix in the prismatic layer [26, 27]. KRMP was shown to exist in the insoluble matrix. Therefore, we conducted the chitin-binding assay of rKRMP-3, rBR and rGYR, with GST as the control protein. GST and rBR were washed through with saline and no band was detected in the subsequent washings. In contrast, rKRMP-3 and rGYR were not eluted with saline and the acidic solution, but did elute with the detergent solution (Fig 4). The results showed that KRMP-3 could bind to chitin and the ability was due to the GY region.

Proteins containing Rebers-Riddiford (RR) [28] consensus sequences have been shown to bind chitin *in vitro* [16, 29]. In arthropod cuticles, the Rebers-Riddiford motif is most widely spread in chitin-binding proteins [30]. However, in mollusks, it was found that proteins without the RR sequences also showed chitin-binding ability *in vitro*. For example, prismalin-14 binds to chitin because of its GY-rich region. The GYR of KRMP and other matrix proteins is also named a RLCD (repetitive, low-complexity domain). RLCDs are found in eggshells and cuticles, suggesting that RLCDs have a key role to play in structural construction [12] and this may be the reason why these structures show good mechanical toughness [31, 32]. In our study, the GYR of KRMP-3 was shown to bind to chitin, which provided direct evidence of the function of an RLCD. The chitin-binding domains of chitinase are known to form a β-sheet structure to bind chitin [26]. From knowledge about matrix proteins, the GY region of prismalin-14 and KRMP-3 may possibly be cross-linked by quinone-tanning to form β-sheets. It is possible that MSI31 [33], prismalin-14 [18, 34], prisilkin-39 and the shematrin family [32], also with Gly-rich sequences such as GGY, are involved in the formation of the prismatic layer in the same way (i.e., β-sheet structure).

### Morphology of the calcite crystals induced by rKRMP-3, rBR and rGYR *in vitro*

**Fig 5:**
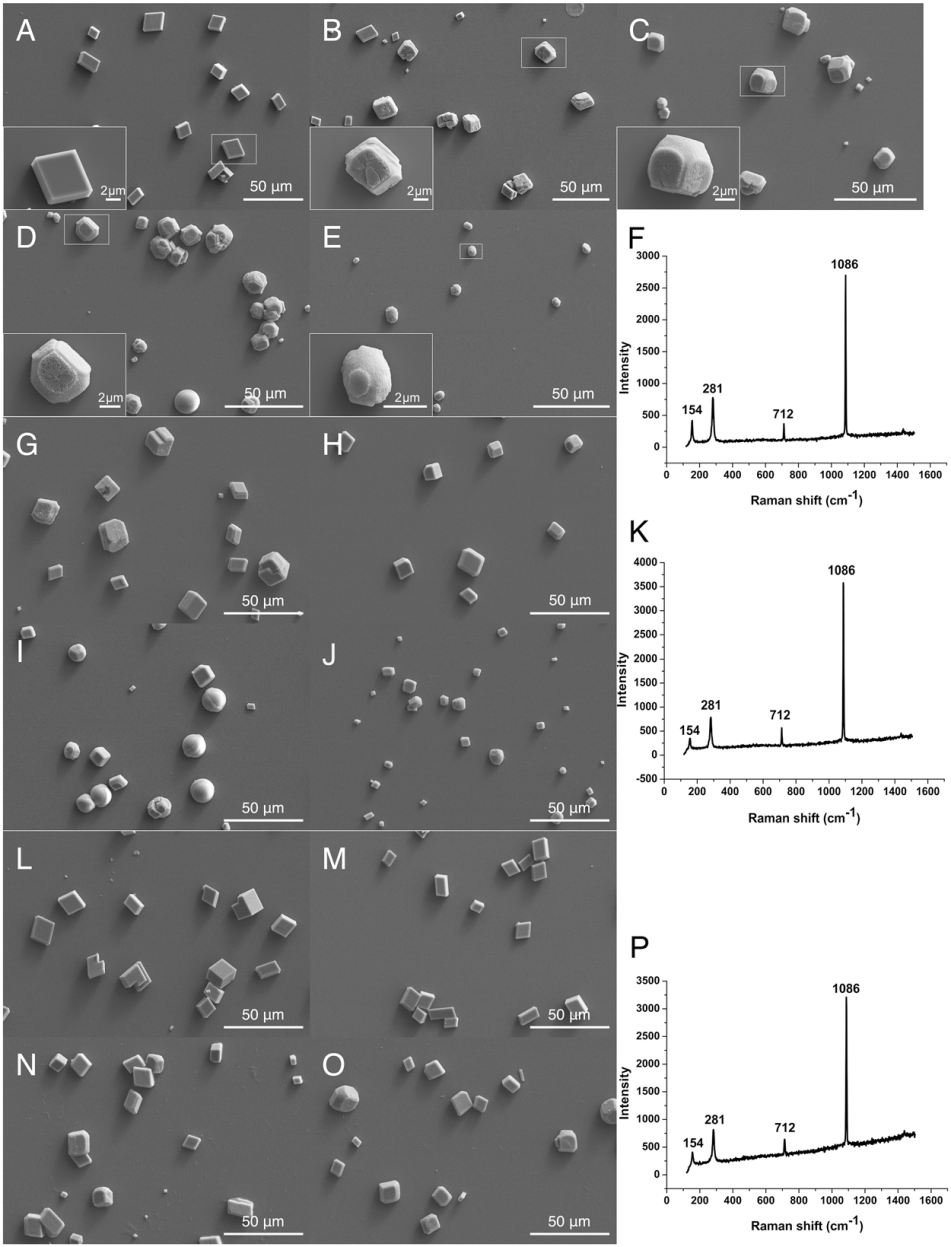
SEM images of *in vitro* crystallization of calcite in the presence of rKRMP-3, rBR, rGYR and Raman spectrum analysis of crystals. Crystals were grown in the presence of 40 μg/ml GST (A), 2 μg/ml rKRMP-3 (B), 10 μg/ml rKRMP-3 (C), 20 μg/ml rKRMP-3 (D), 40 μg/ml rKRMP-3 (E), 2 μg/ml rBR (G), 10 μg/ml rBR (H), 20 μg/ml rBR (I), 40 μg/ml rBR (J) and 2 μg/ml rGYR (L), 10 μg/ml rGYR (M), 20 μg/ml rGYR (N), 40 μg/ml rGYR (O). The crystals in left bottom of the image are enlarged images of the boxed part, respectively. F, K, P showed the raman spectra of the crystals from 40 μg/ml rKRMP-3 (E), 40 μg/ml rBR (J), 40 μg/ml rGYR (O), respectively.

The interactions of rKRMP-3, rBR and rGYR with the growth of calcites were examined. Generally, the biominerals can be divided into angular, sub angular, sub rounded, rounded according to the shapes. In our experiments, we found that the shapes of crystals changed with the addition of rKRMP-3, rBR and rGYR. In a negative control experiment, 40 μg/ml GST was added, and the induced crystals were typically angular with smooth surface (Fig 5A). The average length and width were 13.27 μm and 10.92 μm, respectively. At 2 μg/ml of rKRMP-3, the morphology of the calcite crystals changed. All crystals exhibited rounded edges and steps that could be visualized (Fig 5B). With increasing amount of rKRMP-3 (10–40 μg/ml), the calcite gradually changed from angular to round (Fig 5C–5E). At 40 μg/ml of rKRMP-3, most calcites obtained were round crystals and their size became smaller (Fig 5E). Moreover, the morphology of the crystals gradually changed in the system containing rBR (Fig 5G–5J) and rGYR (Fig 5L–5O). In order to quantify the shapes and sizes of crystals in a well appropriately, we observed them at low magnification (S2 Fig). At 40 μg/ml of rKRMP-3, 97.3% of the formed crystals were round with an average diameter of 11.82 μm approximately. At 40 μg/ml of rBR, sub round and round crystals took up 70.3% of the crystals, with an average diameter of 10.01 μm approximately. At 40 μg/ml of rGYR, sub round and round crystals took up 33.8% of the crystals, with an average diameter of 12.36 μm approximately. The Raman spectrum analysis of the obtained crystals from the three groups (E, J, O) suggested that all of them were calcite, with absorption peaks at 281, 712 and 1086 cm^–1^, respectively (Fig 5F, 5K and 5P).

*In vitro* studies have shown that natural and recombinant matrix proteins control the crystal morphology [16, 34–39]. Lysine residues, which are abundant in the KRMP BR region, have been implicated in controlling crystal formation in biomimetic silicification experiments previously [40–42]. In addition, the presence of two lysine-rich peptides were found to produce circular morphologies in CaCO_3_ precipitation experiments [27]. Moreover, the prismatic layer was shown to disintegrate the calcitic prisms after KRMP genes were knocked down following dsRNA injection [43]. In our studies, the dose effect of rKRMP-3 on the growth of calcite showed that the morphology of calcite crystals changed from 2–20 μg/ml; the crystal edges gradually disappeared. The crystals became round and crystal sizes became smaller with higher concentrations of KRMP-3 (40 μg/ml). Moreover, the system containing rBR showed similar effects of crystal morphology as observed in the rKRMP-3 system, whereas the crystals in the system with rGYR showed even minor changes. In total, our results are consistent with the previous reports.

### Morphology of the aragonite crystals induced by rKRMP-3 *in vitro*

**Fig 6:**
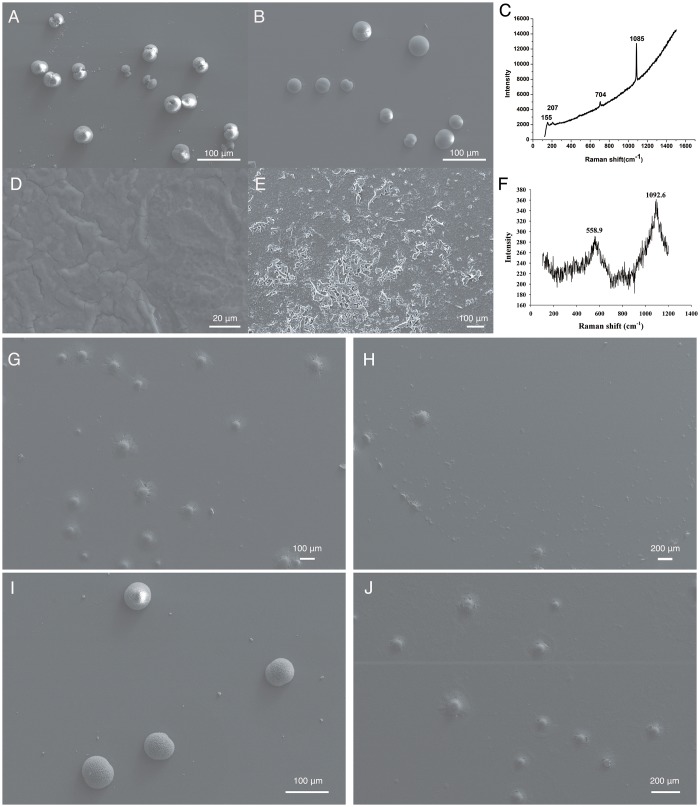
SEM images of *in vitro* crystallization of aragonite in the presence of rKRMP-3, rBR, rGYR and Raman spectrum analysis. Crystals were grown in the presence of 50 mM MgCl_2_ with 10 μg/ml GST(A), 2 μg/ml rKRMP-3 (B), 10 μg/ml rKRMP-3 (D, E) and 2 μg/ml rBR (G), rGYR (I), 10 μg/ml rBR (H), rGYR (J). Raman spectra showed the typical crystals formed in A, B, I were aragonite, with the characteristic peaks at 207, 704, and 1085 cm^-1^ (C). Raman spectra of the material precipitated in the presence of 10 μg/ml rKRMP-3 (C, D) showed the absorption peaked at 558.9 and 1092.6 cm^–1^ (F).

The presence of Mg^2+^ in the calcium carbonate solutions favors the formation of aragonite [37], so Mg^2+^ (50 mM) was added to the aragonite precipitation system. In the control group, GST served as a negative control, with the formation of round-shaped crystals (Fig 6A). At 2 μg/ml of rKRMP-3, no significant effect on the crystals was observed (Fig 6B). At 10 μg/ml of rKRMP-3, no shaped crystals were observed in the field of vision but the material precipitated like a film (Fig 6D and 6E). Furthermore, we examined the film-material with Raman spectra. Raman measurements showed that the absorption peaked at 558.9 and 1092.6 cm^–1^, not corresponding with any kinds of crystals (Fig 6F). Together, there were no shaped crystals in the well, and we couldn’t define the material.

At 2 μg/ml of rBR, no typical crystals were found (Fig 6G) and a few crystals formed with 10 μg/ml of rBR (Fig 6H). However, typical crystals formed with 2 μg/ml of rGYR (Fig 6I). Only at 10 μg/ml of rGYR, no typical crystals were displayed (Fig 6J). Raman measurements showed that the observed typical crystals formed in Fig 6A and 6B, I were aragonite (Fig 6C). As for the aragonite growth *in vitro*, the dose effect of rKRMP-3 showed strong inhibition. Compared with rKRMP-3, rBR showed stronger inhibition of the formation of aragonite; even at 2 μg/ml of rBR no typical crystals were found. rGYR was found to show weaker inhibition of aragonite crystallization when compared with the activity of rBR and rKRMP-3.

### *In vitro* inhibitory activity of rKRMP-3, rBR and rGYR on calcium carbonate precipitation

**Fig 7:**
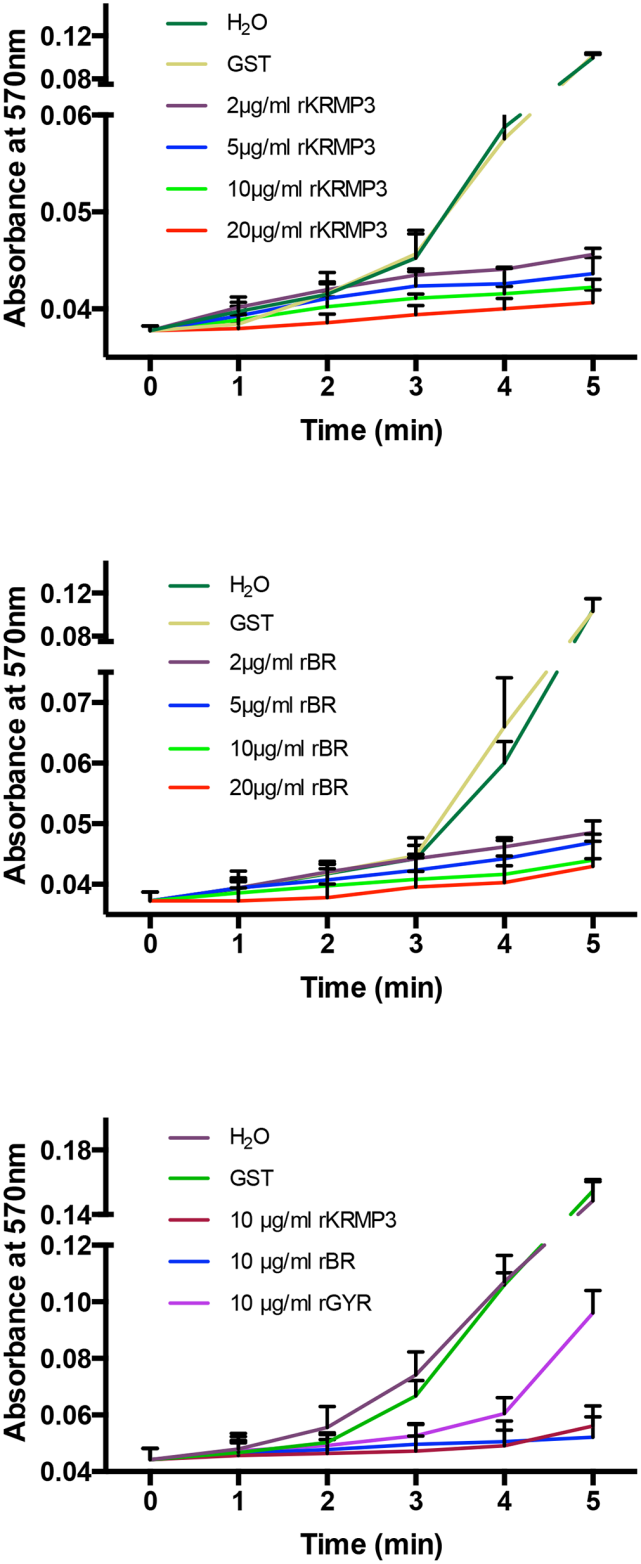
*In vitro* inhibition of CaCO_3_ precipitation of by rKRMP-3 (A), rBR (B) and rGYR. 2 μg/ml rKRMP-3 and rBR is effective to inhibit the precipitation and the inhibitory activity is dose-dependent. Inhibitory activity of 10 μg/ml rKRMP-3 is almost the same as 10 μg/ml rBR, while the activity of 10 μg/ml rGYR is lower (C). 20 μg/ml GST had no effect on the precipitation of calcium carbonate.

The effect of rKRMP-3 and its two domains on calcium carbonate precipitation was examined *in vitro*. In the blank experiments, the absorbance value at 570 nm, which corresponds to the precipitation, increased and reached ~0.1 in 5 min. When rKRMP-3 was added, the variation drastically changed. The highest absorbance value did not exceed 0.045 with the addition of 2 μg/ml of rKRMP-3. The absorbance at every minute reduced as the amount of rKRMP-3 present increased. The same effect was found when rBR was added to the system. rKRMP-3 and rBR inhibited calcium carbonate precipitation dose-dependently and almost completely inhibited this precipitation at ~20 μg/ml (Fig 7A and 7B). To examine the contribution of the two regions to the inhibitory ability, 10 μg/ml rKRMP-3, rBR and rGYR were added to the system, respectively. The inhibitory effect of rKRMP-3 and that of rBR was almost identical, whereas the inhibitory effect of rGYR was weaker (Fig 7C). In contrast, GST had no effect on the precipitation of calcium carbonate. The results showed that rKRMP-3 significantly inhibited CaCO_3_ precipitation, changed the morphology of calcite and inhibited the growth of aragonite *in vitro*.

The results of the inhibitory activity on calcium carbonate precipitation by rKRMP-3 and rBR could explain the effect on the growth of crystals. The observation that rKRMP-3 displayed growth control of calcite and inhibition of aragonite is consistent with the shell localization. The matrix protein from the prismatic layer inhibited the growth of aragonite and controlled the growth of calcite. In molluscan shell formation, crystal nucleation and growth inhibition are two antagonistic mechanisms. KRMP-3 should have an effect on growth inhibition. This result resembles that of prisilkin-39 and differs from that of most acidic matrix proteins. The functionalities identified are due to a lysine-rich basic region of KRMP-3, which may interact with the carbonate (or bicarbonate) ions during formation of the shell. Additionally, the lysine side chains can be modified to participate in intermolecular cross-linking via Schiff’s base conjugates [9]. In the case of KRMP-3, the lysine-rich region is also postulated to interact with acid proteins or the anionic groups of sugars in the matrix polysaccharides and glycoproteins, which would be in addition to the interaction with carbonate (or bicarbonate) ions.

### Multiple sequence alignment of the KRMP

**Fig 8:**
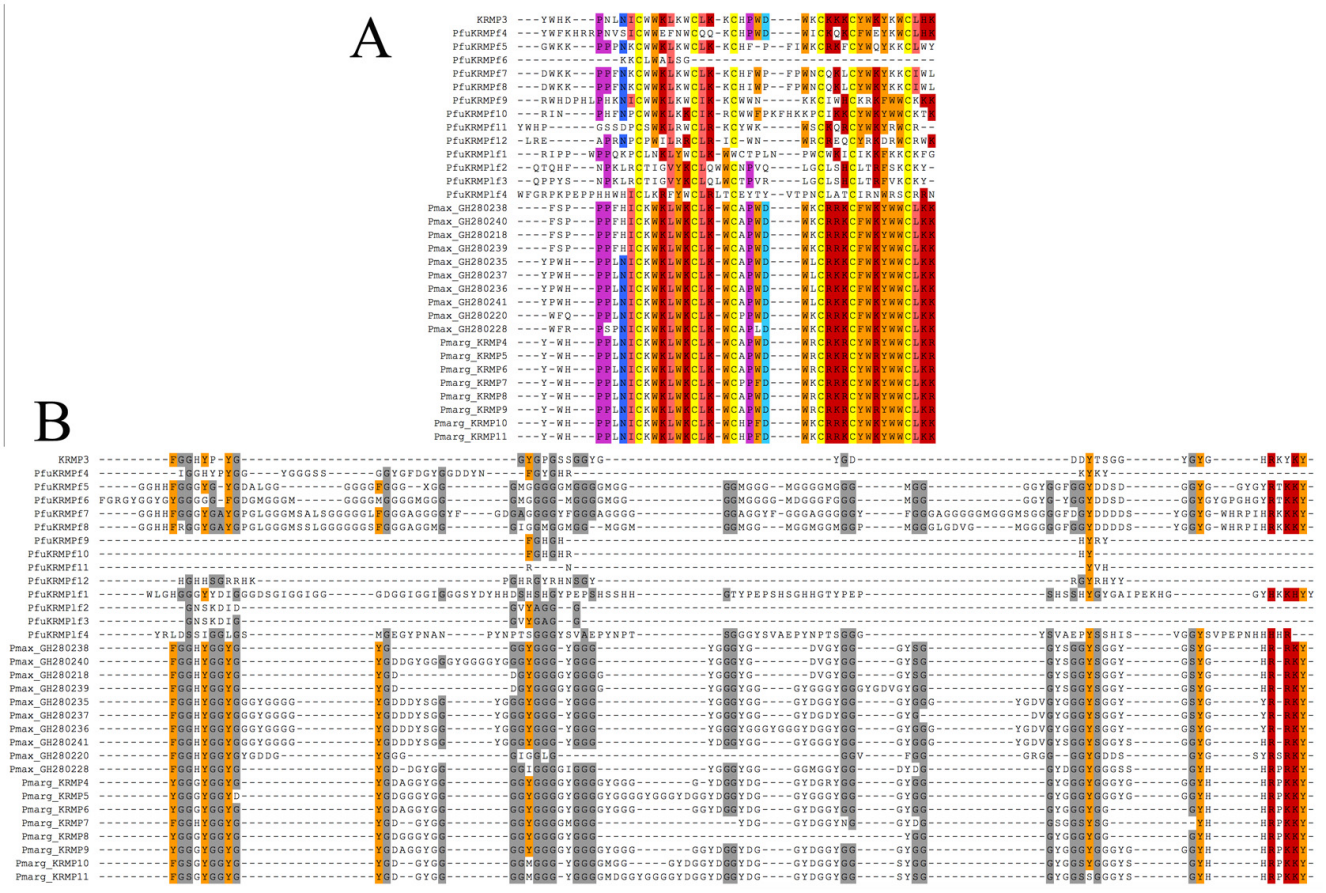
Multiple sequence (except for signal peptide) alignments of KRMP-3 from *P*. *fucata* with members from *P*. *margaritifera* and *P*. *maxima*. They all have two functional regions, a lysine-rich region (A) and a Gly/Tyr-rich region (B). GenBank Accession Numbers of KRMP-4 to KRMP-6 in *P*. *maxima* are EF183517 to EF183519, and KRMP-7 to KRMP-11 are EF192240 to EF192244. GenBank Accession Numbers of KRMPs in *P*. *margaritifera* are from GH280235 to GH280241 and GH280218, GH280220 and GH280228.

According to our results on KRMP-3, we predict that KRMP-1 and KRMP-2 may also share similar characteristics and functions, as the deduced amino acid sequences of KRMP-3 and KRMP-2 are almost identical except for residue 74th, and the sequences of KRMP-1 and KRMP-3 (or KRMP-2) show more than 80% identity. Additional 13 new *P*. *fucata* KRMP sequences (pfuKRMPf4 to pfuKRMPf12 and pfuKRMPlf1 to pfuKRMPlf4) were reported afterwards [12]. Meanwhile, the members of the KRMP family from *P*. *maxima* and *P*. *margaritifera* were reported [11, 13]. Except for the signal peptide, these proteins all possess the same functional regions as KRMP-3, a lysine-rich region and a Gly/Tyr-rich region (Fig 8). The sequence alignments between KRMP-3 and other members from three pearl oysters reveal that their lysine-rich regions share a higher similarity, whereas the similarity of their GYRs is lower. The deduced amino acid sequence alignments of the lysine-rich regions between KRMP-3 and other members in *P*. *fucata* indicate: KRMP3 and KRMPf4 to f8 share highest similarity from 68% to 71%, whereas KRMP-3 and KRMPf9 to f12 show 50–62% similarity, KRMP-3 and KRMPlf1 to lf4 only have 26% to 38% similarity. These results were consistent with phylogenetic analysis of KRMPs, indicating that KRMPf4 to f8 were *P*. *fucata* specific KRMPs, KRMPf9 to f12 were early divergent KRMPs, and KRMPlf1 to lf4 were KRMP-like proteins [12]. Moreover, the alignments between KRMP-3 and *P*. *margaritifera* KRMP-4 to 11 reveal that their lysine-rich regions share a higher similarity around 82%, whereas the similarities between KRMP-3 and members from *P*. *maxima* differ from 71% to 79%. Together, higher similarity of the lysine-rich regions among these proteins indicates they may play the same roles in shell formation. Also some proteins similar to KRMPs were found in the *Mytilus edulis* mantle tissue, suggesting that KRMPs are not limited to *Pinctada* [44]. KRMP may be a pivotal matrix protein family in mollusk. Therefore, the clarification of KRMP-3 lays the foundation for understanding the function of KRMP family, while further functional research of them is required.

## Conclusions

KRMP-3 is localized on the organic sheet and the prismatic sheath and is shown to be a component of the insoluble matrix. The ability of KRMP-3 to bind chitin meets the demands for proteins localized in those areas of the shell. The changes in the morphology of calcite and the inhibition of aragonite crystal growth reveal the possible functions of KRMP-3 in the control of related crystallization phases in shell formation. The functional rBR region is found to participate in the growth control of crystals and the rGYR region is bound to chitin; both attributes are functional characteristics of KRMP-3.

## Supporting Information

S1 Fig Immunofluorescence localization of native KRMP-3 from multiple specimens of *P*. *fucata*. (PDF)

S2 Fig SEM images of *in vitro* crystallization of calcite in the presence of rKRMP-3, rBR, rGYR at low magnification. (PDF)
