# Supplementary material for: Dual Roles of the Lysine-Rich Matrix Protein (KRMP)-3 in Shell Formation of Pearl Oyster, Pinctada fucata
Source: PLoS One. 2015 Jul 10;10(7):e0131868. doi: 10.1371/journal.pone.0131868 (PMC4498902; doi:10.1371/journal.pone.0131868)

Supporting information

S1 Fig. Immunofluorescence localization of native KRMP-3 from multiple specimens of *P. fucata*. A-D, the white arrows showed positive signal in the organic sheet and black arrows showed positive signal in the prismatic sheath from four different shells. E-H, negative control staining with preimmune serum. F, H are enlarged images of the E, G, respectively. The white arrows in E-H indicated only a small amount of background staining were detected. P, prismatic layer; N, nacreous layer; OS, organic sheet. Scale bars in (A, B, E, G), 12.5 μm and in (C, D, F, H), 50 μm.

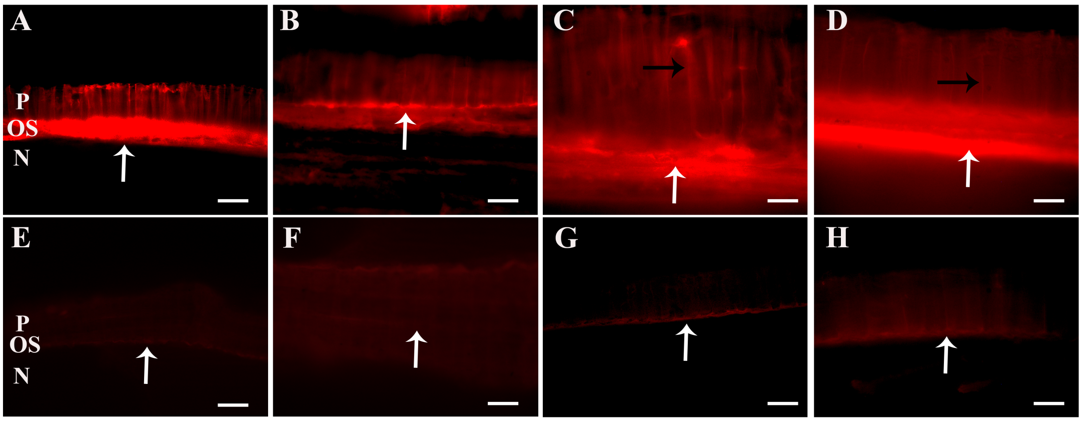

Supplement: S1 Fig — (PDF) [file pone.0131868.s001.pdf]
