# Supplementary material for: Dual Roles of the Lysine-Rich Matrix Protein (KRMP)-3 in Shell Formation of Pearl Oyster, Pinctada fucata
Source: PLoS One. 2015 Jul 10;10(7):e0131868. doi: 10.1371/journal.pone.0131868 (PMC4498902; doi:10.1371/journal.pone.0131868)

Supporting information

S2 Fig. SEM images of *in vitro* crystallization of calcite in the presence of rKRMP-3, rBR, rGYR at low magnification. Crystals were grown in the presence of 40 µg/ml GST (A), 40 µg/ml rKRMP-3 (B), 40 µg/ml rBR (C) and 40 µg/ml rGYR (D). Scale bars, 100 µm.

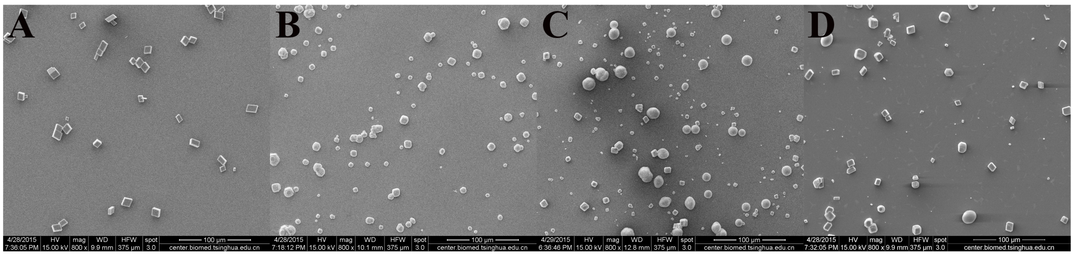

Supplement: S2 Fig — (PDF) [file pone.0131868.s002.pdf]
